# Patient-ventilator asynchrony as a predictor of weaning failure in mechanically ventilated COPD patients

**DOI:** 10.1186/s43168-021-00076-9

**Published:** 2021-06-03

**Authors:** Samiaa H. Sadek, Maha M. El-kholy, Marwa S. Abdulmoez, Reham M. El-Morshedy

**Affiliations:** Department of Chest Diseases and Tuberculosis, Faculty of Medicine, Assiut University Hospital, El-Gamaa St. Assiut, Assiut, 71111 Egypt

**Keywords:** Mechanically ventilated, Weaning failure, COPD, Asynchrony

## Abstract

**Background:**

Patient-ventilator asynchrony is a common problem in mechanically ventilated patients. It is associated with adverse effects including increased work of breathing, patient discomfort, increased need for sedation, prolonged mechanical ventilation, weaning difficulties, and weaning failure. The purpose of the present was to describe patient-ventilator asynchrony and its impact on weaning outcomes in mechanically ventilated chronic obstructive pulmonary disease (COPD) patients.

**Results:**

One hundred mechanically ventilated COPD patients were enrolled in this prospective study. Weaning failure (need of NIV or reintubation within 48 h) was noticed in 27 (27%) patients while 73 (73%) patients had successful weaning. Patients with failed weaning had significantly higher asynchrony index (A.I) and ineffective trigger index (ITI) in comparison with those with successful weaning (7.69 ± 3.71, 3.46 ± 2.59 versus 6.27 ± 3.14, 2.47 ± 2.08, respectively; *P* value< 0.04). Data were expressed as mean ± standard deviation.

**Conclusion:**

High asynchrony index and high ineffective trigger index may be early predictors of weaning failure in mechanically ventilated COPD patients.

## Background

Patient-ventilator asynchrony is defined as a lack of organization between the patient and ventilator timing of both inspiration and expiration [1]. It is a commonly reported problem during mechanical ventilation. Thille et al. found that 24% of patients developed asynchrony in at least 10% of their breaths; moreover, they stated that the most frequent asynchronies were ineffective triggering and double triggering [2]. Eighty percent of chronic obstructive pulmonary disease (COPD) patients experienced ineffective triggering, where it is considered the most frequent asynchrony in this group of patients [3–5]. Asynchrony between patient and ventilator also leads to prolonged duration of invasive ventilation and increased possibility of weaning failure [6].

Both spread and intensity of asynchrony during the early phase of weaning in COPD patients have never satisfactorily been described. The aim of the current study was to describe the patient-ventilator asynchrony and its impact on weaning outcomes in mechanically ventilated COPD patients.

## Methods

The current prospective observational cohort study was performed in respiratory intensive care unit (R.I.C.U), Chest Department, Assuit University Hospital. The study was performed during the period between February 2018 and August 2019; it involved 100 mechanically ventilated COPD patients. All patients recruited in this study were diagnosed with COPD according to the GOLD guideline criteria (based on history, physical examination, chest radiography, and previous pulmonary function tests if available). If not available once the patient became stable, spirometry was done to confirm the diagnosis [7]. The exclusion criteria included age < 18 years, tracheostomy, failure to trigger breaths even in cases of receiving neuromuscular blocking agents, encephalopathy which is not caused by hypercapnia or hypoxemia either post-arrest or due to cerebrovascular stroke, patients with unplanned weaning, and COPD patients where intubation is not related to exacerbation, e.g., acute pulmonary edema.

Baseline characteristics including age, gender, smoking history, history of the present illness, and the severity of illness were measured by Acute Physiology and Chronic Health Evaluation II (APACHE II) [8].

Arterial blood gasses were obtained on admission (named baseline) immediately before intubation, 24 h after intubation, during spontaneous breathing trial (SBT), 2 h after weaning, 24 h after weaning, and when there is a clinical demand. Clinical and ventilator data were recorded. The duration of sedation and also the duration of mechanical ventilation prior to inclusion in the study were determined.

### Equipment utilized

All patients underwent mechanical ventilation using a Puritan Bennett 840 (Puritan Bennett, Pleasanton, CA). Patients underwent recording of pressure, flow, and volume waveforms for a period of 30 min. Ventilator settings were set by clinicians caring for the patient and were not modified during the 30-min observation period.

### Patient-ventilator asynchrony

Patient-ventilator asynchrony is detected in 30-min sessions at 12, 24, 36, and 48 h following intubation. The asynchrony index by visual assessment of pressure, flow, and volume graphs is detected. Asynchrony index (AI) is calculated by dividing the number of asynchronies by the total respiratory rate (ventilator cycle + wasted effort) × 100. Ineffective trigger index (ITI) is calculated in the same way by dividing the number of ineffective triggers by the total respiratory rate (ventilator cycle + wasted effort) × 100 [2]. An AI of 10% or higher defined severe patient-ventilator asynchrony [2].

### Type of asynchronies

#### Trigger asynchrony

Ineffective triggering or trigger asynchrony means ineffective effort where the ventilator is unable to detect patient effort. It is characterized by the inability to deliver ventilator breath despite the change in expiratory flow, or decline in the waveform of pressure/time [2].

#### Double triggering

Double triggering is characterized by two subsequent inspirations with short expiratory time, where the first cycle is being patient triggered [9, 10].

#### Auto-triggering

Auto-triggering means that breath is delivered from the ventilator without patient triggering. It is observed as a cycle that is delivered to the patient in the absence of a prior decrease in airway pressure [2].

#### Flow asynchrony

Flow asynchrony implies that the delivery of a mechanical breath is not suitable to the needs of the patient. One can observe the concave pressure-time airway curve of the assisted breath as a result of inadequate flow delivery; this means the flow provided by the mechanical breath is lower than the patient demand [11].

#### Cycle asynchrony

##### Premature cycle

The neural inspiratory time is longer than mechanical inspiratory time, and it is presented as a significant airway pressure decrease detected immediately following the end of the inspiratory phase [11].

##### Late cycle

The neural inspiratory time is shorter than the mechanical inspiratory time. It is characterized by an abrupt decrease in inspiratory flow in the flow/time curve and an increase in airway pressure near the end of the inspiratory phase [11].

### Weaning from mechanical ventilation

Weaning trial was considered after patient stabilization. Patients underwent daily assessment of readiness to wean. Weaning was done according to the followed protocol in R.I.C.U [12] (using the spontaneous mode of weaning with low-pressure support (8 cm H_2_O) and zero PEEP with the same FIO_2_ (< 40%) for at least 2 h).

The study design was approved by the Scientific Ethics Committee of the Faculty of Medicine of Assiut University. After meeting the inclusion criteria, informed written consent was obtained from the surrogate decision-maker before enrollment.

### Statistical analysis

Data analysis was done using SPSS (Statistical Package for Social Science for Windows statistical package, version 16.0) [13]. The results of different variables were presented as mean ± SD. The mean ± SD values were compared between the groups using Student’s t test or the Mann-Whitney test depending on whether the distribution deported from the normal. Logistic regression analysis was used to demonstrate the association of different variables to the failed group, expressed as the odds ratios (OR) and 95% confidence intervals (CI) for values showing statistical significance or tendency toward significance in Student’s t test. A p-value < 0.05 was considered statistically significant.

## Results

**Fig. 1:**
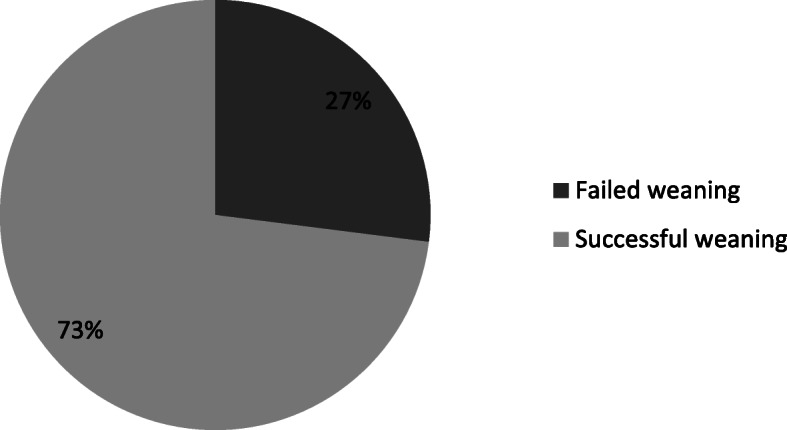
Weaning outcome in studied patients

**Fig. 2:**
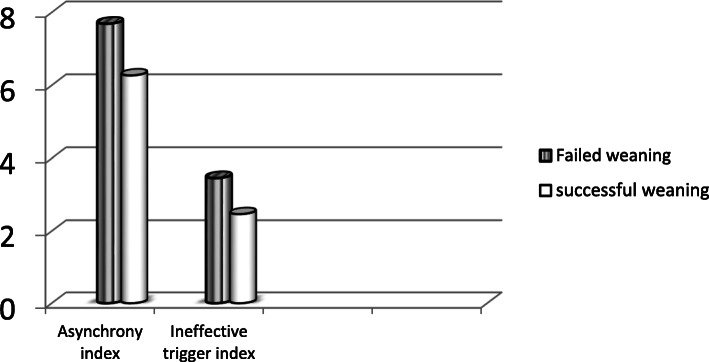
Asynchrony index and ineffective trigger index in studied patients based on the weaning outcome

A total of 100 mechanically ventilated COPD patients were enrolled in this study. Failed weaning was noticed in 27 (27%) patients while 73 (73%) patients had successful weaning as shown in Fig. 1. The main characteristics of the patients are indicated in Table 1. There were no significant differences between both groups with respect to age and sex. Smoking index was significantly higher among those with failed weaning (1400 (300–2400) vs. 800 (250–2000); *P* = 0.02). APACHE score was found to be significantly higher among those with failed weaning (29.01 ± 5.17 vs. 24.50 ± 4.19; *P* < 0.001). Regarding the duration of mechanical ventilation, it was significantly higher among those with failed weaning (9.70 ± 3.94 vs. 4.26 ± 1.23; *P* < 0.001). Clinical and ventilator data of the studied patients at the time of weaning showed significant differences between the two groups of patients where respiratory rate, peak pressure, rapid shallow breathing index, level of PEEP applied, and also the duration of sedation were higher among patients with failed weaning, whereas spontaneous tidal volume and static and dynamic compliance were higher among patients with successful weaning. Table 2 shows the patients’ asynchronies in the studied patients based on the outcomes of weaning. Patients with failed weaning had significantly higher ineffective trigger, double trigger, delayed trigger, and total asynchronies in comparison with those with successful weaning (*P* < 0.05). In particular, patients with failed weaning had significantly higher asynchrony index (A.I) and ineffective trigger index (ITI) in comparison with those with successful weaning (7.69 ± 3.71, 3.46 ± 2.59 versus 6.27 ± 3.14, 2.47 ± 2.08, respectively; *P* value 0.04) (Fig. 2). Both groups had insignificant auto-trigger, early cycle, and flow asynchrony (*P* > 0.05).

**Table 1:** Baseline data of studied patients based on weaning outcome

	Successful weaning (*n* = 73)	Failed weaning (*n* = 27)	*P* value
Age (years)	61.72 ± 6.79	63.74 ± 8.70	0.22
Sex			
Male	57 (78.1%)	18 (66.7%)	0.18
Female	16 (21.9%)	9 (33.3%)	
Smoking index	800 (250–2000)	1400 (300–2400)	0.01*
APACHE	24.50 ± 4.19	29.01 ± 5.17	**< 0.001** *
Duration of mechanical ventilation prior to inclusion, days	4.26 ± 1.23	9.70 ± 3.94	< 0.001*
Clinical and ventilator data			
RR (cycle/min)	22.32 ± 4.04	31.51 ± 7.19	< 0.001*
STV (mm^3^)	383.78 ± 89.55	295.48 ± 67.86	< 0.001*
Minute ventilation (%)	8.01 ± 1.97	7.70 ± 2.13	0.50
Peak airway pressure (mmHg)	28.28 ± 4.46	30.51 ± 5.82	**0.04**
PEEP cm H_2_O	4.65 ± 1.57	5.77 ± 1.22	< 0.001*
RSBI	71.08 ± 20.24	124.92 ± 38.32	< 0.001*
Static compliance	46.11 ± 11.56	33.40 ± 8.25	< 0.001*
Dynamic compliance	37.42 ± 7.83	28.18 ± 6.37	< 0.001*
Duration of sedation (days)	1.16 ± 0.38	3.18 ± 1.05	< 0.001*
PaO_2_/FiO_2_, mmHg	**226 ± 5.2**	**228 ± 6.3**	**0.45**
PaCO_2_, mmHg	**58.83 ± 8.5**	**63.73 ± 10.70**	**0.47**
PaO_2_	**71.15 ± 7.73**	**72.63 ± 8.16**	**0.41**

*RR* respiratory rate, *STV* spontaneous tidal volume, *RSBI* rapid shallow breathing index, *PEEP* positive end-expiratory pressure

**Table 2:** Patients’ asynchronies in studied patients based on the outcome of weaning

Type of asynchrony	Successful weaning (*n* = 73)	Failed weaning (*n* = 27)	*P* value
Ineffective trigger	16.89 ± 10.60	30.81 ± 23.18	< 0.001*
Double trigger	4.76 ± 3.79	6.25 ± 4.46	0.02*
Auto trigger	1.71 ± 0.75	2.34 ± 1.55	0.06
Delayed cycle	11.79 ± 7.40	15.48 ± 7.57	0.03*
Early cycle	8.08 ± 7.47	10.81 ± 8.97	0.07
Flow asynchrony	2.72 ± 0.98	3.60 ± 2.03	0.12
Total asynchronies	43.27 ± 20.27	70.11 ± 32.51	< 0.001*

**Fig. 3:**
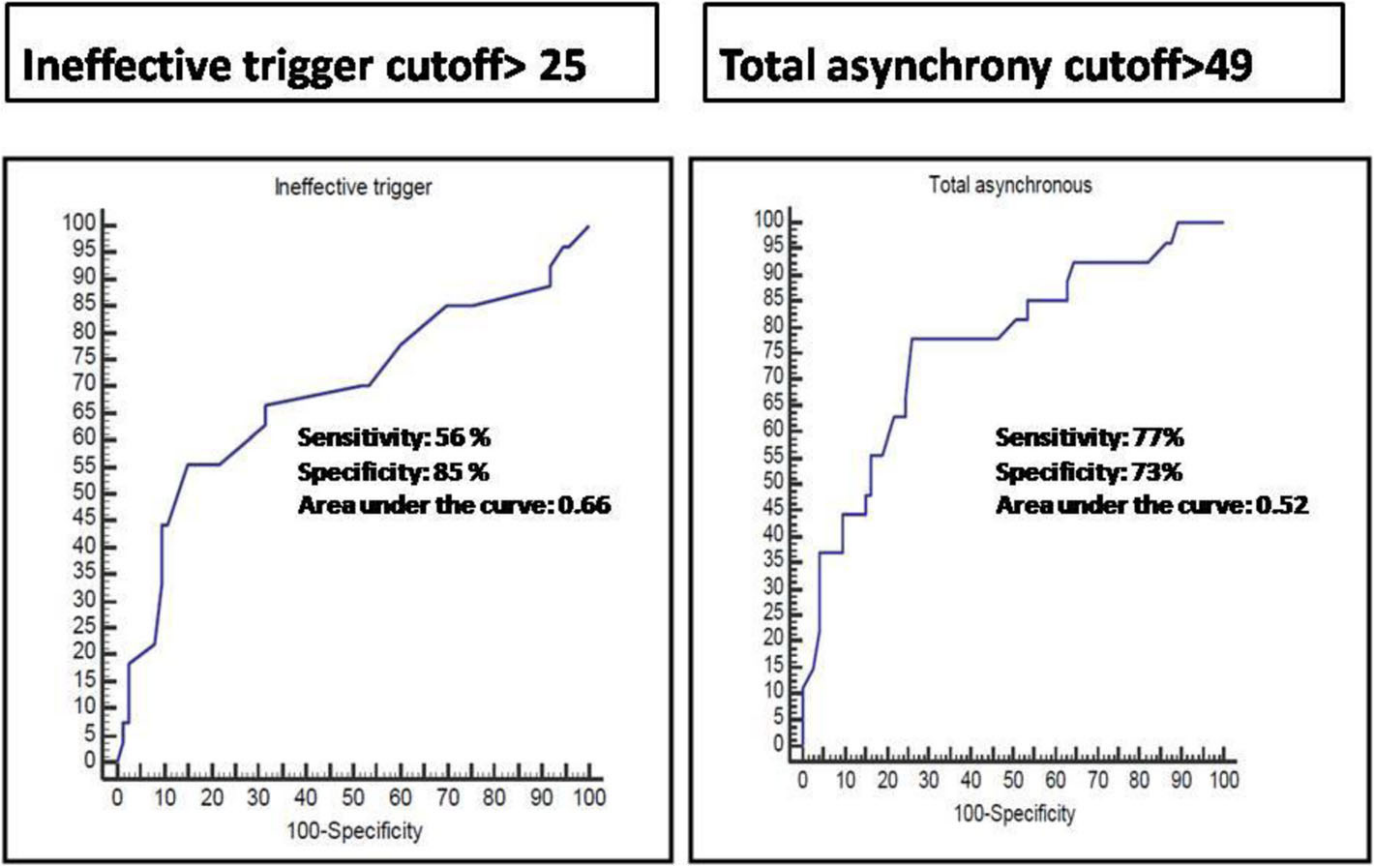
Sensitivity and specificity of an ineffective trigger and total asynchrony on predicting weaning failure

Multivariate regression analysis showed that both ineffective trigger and total asynchronies were reliable predictors for failed weaning. At a cutoff of > 25, ineffective trigger had 56% sensitivity and 85% specificity with an area under the curve of 0.66 for the prediction of failed weaning. The total asynchronies had 77% sensitivity and 73% specificity with an area under the curve of 0.52 for the prediction of failed weaning at a cutoff of > 49 (Fig. 3).

## Discussion

Librating the patient from endotracheal tube and mechanical ventilation with subsequent successful weaning is the main target since the time of initiating invasive mechanical ventilation. Several studies assessed patient-ventilator asynchrony during mechanical ventilation and its impact on the prolonged duration of mechanical ventilation [3, 5, 6]. The present study assesses the types of patient-ventilator asynchrony in COPD patients and their impact on weaning outcomes.

With respect to the baseline data of the studied patients, smoking index, APACHE II score, and duration of mechanical ventilation were significantly higher in failed weaning COPD patients compared to the successful weaning group. A higher smoking index may lead to more deterioration of pulmonary function, and increased disease severity and systemic inflammation. APACHE II score reflects the severity of illness of the primary disease and its effect on weaning outcomes. Several studies documented higher APACHE II scores in failed weaning patients [14, 15]. Also, longer duration of mechanical ventilation in the failed weaning group may reflect increased disease severity which is supported by the presence of a higher APACHE II score and more significant respiratory muscle weakness. The present study observed higher RSBI in failed weaning COPD patients; actually, several studies in COPD and non-COPD patients reported higher levels of RSBI in the failed group of patients, but they reported different cutoff values for RSBI [14, 16]. The most commonly reported cutoff value for RSBI was > 105, but as regards COPD because of the presence of ineffective respiratory effort with the subsequent failed triggering of ventilator leading to inaccurate assessment of RSBI [17]. Another study reported that RSBI < 80 predicts failed weaning in 56% of mechanically ventilated COPD patients [5]. Static and dynamic compliance were higher among patients with successful weaning in the current study. The changes in respiratory mechanics of COPD patients in the form of airway obstruction, hyperinflation, increased respiratory rate, and subsequent dynamic hyperinflation, all of these events direct the functional residual capacity to the flat portion of the P-V curve leading to increased work of breathing and decreased compliance [18]. Studies documented that the presence of COPD per se is a predictor of weaning failure [18, 19]. Ghiani et al. [20] supported the present study; they documented that patients with failed weaning had lower compliance and increased work of breathing.

With respect to the patient-ventilator asynchrony, the present study demonstrated that patients with failed weaning had significantly higher ineffective trigger, double trigger, delayed trigger, and total asynchronies in comparison with those with successful weaning (*P* < 0.05). In particular, patients with failed weaning had significantly higher asynchrony index (A.I) and ineffective trigger index (ITI). De Wit et al. [21] observed that ineffective trigger is frequently observed during mechanical ventilation, which participated in weaning failure, prolonged ICU stay, and morbidity. Also, several studies attributed asynchronies in ventilated COPD patients to abnormal respiratory mechanics, where patients with hyperinflation and intrinsic positive end-expiratory pressure PEEP had a higher prevalence of ineffective trigger [5, 22]. The present study demonstrated higher level of PEEP is needed in the failed weaning group which may be attributed to the presence of a higher level of auto-PEEP and hyperinflation in this group of patients. Grasso et al. and De Wit et al. [21, 23] also attributed ineffective triggering to sedation which impairs respiratory drive, the result which agrees with the present study where failed weaning patients had a significantly longer duration of sedation. Similarly, other studies demonstrated that all types of asynchrony are common in COPD patients, and they participated in mortality and length of hospital stay [24]. Multiple studies demonstrated that patients with ineffective trigger index and asynchrony index > 10% had longer duration of mechanical ventilation and ICU and hospital stay [25, 26].

## Conclusion

Patient-ventilator asynchrony is a commonly encountered problem in mechanically ventilated COPD patients. High asynchrony index and high ineffective trigger index may be early predictors of weaning failure in these patients.

## Data Availability

The datasets used and/or analyzed during the current study are available from the corresponding author on reasonable request.

## Abbreviations

COPD: Chronic obstructive pulmonary diseases
NIV: Non-invasive ventilation
APACHE score: Acute Physiologic Assessment and Chronic Health Evaluation score
PEEP: Positive end-expiratory pressure
RSBI: Rapid shallow breathing index
ITI: Ineffective trigger index
AI: Asynchrony index
